# Coupled grain boundary motion in aluminium: the effect of structural multiplicity

**DOI:** 10.1038/srep25427

**Published:** 2016-05-03

**Authors:** Kuiyu Cheng, Liang Zhang, Cheng Lu, Kiet Tieu

**Affiliations:** 1 School of Mechanical, Materials and Mechatronic Engineering, University of Wollongong, Wollongong, NSW 2522, Australia

## Abstract

The shear-induced coupled grain boundary motion plays an important role in the deformation of nanocrystalline (NC) materials. It has been known that the atomic structure of the grain boundary (GB) is not necessarily unique for a given set of misorientation and inclination of the boundary plane. However, the effect of the structural multiplicity of the GB on its coupled motion has not been reported. In the present study we investigated the structural multiplicity of the symmetric tilt Σ5(310) boundary in aluminium and its influence on the GB behaviour at a temperature range of 300 K–600 K using molecular dynamic simulations. Two starting atomic configurations were adopted in the simulations which resulted in three different GB structures at different temperatures. Under the applied shear deformation each GB structure exhibited its unique GB behaviour. A dual GB behaviour, namely the transformation of one GB behaviour to another during deformation, was observed for the second starting configuration at a temperature of 500 K. The atomistic mechanisms responsible for these behaviour were analysed in detail. The result of this study implicates a strong relationship between GB structures and their behaviour, and provides a further information of the grain boundary mediated plasticity in nanocrystalline materials.

Grain boundary (GB) migration is defined as the movement of a grain boundary in perpendicular direction to the boundary plane, and it plays an important role in recrystallization, grain growth, and the plasticity of nanocrystalline (NC) materials. Grain boundary migration can be induced by a gradient of stored plastic deformation energy, boundary curvature, anisotropy of physical properties and applied shear stress^1^,^2^. During the last decade, shear stress induced GB migration has attracted significant research interest because it has been found to be a generic deformation mechanism of NC materials that is related to their exceptional strength and ductility^3^,^4^,^5^,^6^,^7^. Stress-induced GB migration is usually accompanied by grain boundary sliding, which is defined as the relative movement of the two adjacent grains in the direction parallel to the boundary plane. This phenomenon is often called coupled GB motion characterised by a factor β, namely the ratio of the GB sliding speed to the GB migration speed^8^.

The shear stress-induced coupled motion of small-angle grain boundary was first observed experimentally in Zinc by Washburn and Parker^9^ and then in the same material by Li *et al*.^10^. Winning and his co-workers^1^,^11^,^12^ investigated the motion of planar symmetrical and asymmetrical tilt GBs in high-purity aluminium under the influence of external shear stress and found that the migrations of low-angle and high-angle GBs can be induced by the imposed external stress. This phenomenon has recently been investigated in experiments using bicrystals^13^,^14^,^15^, NC materials^3^,^6^, and coarse grained polycrystalline materials^16^,^17^. Gleiter^18^ proposed a step model to explain the migration mechanism of grain boundary, from which it was assumed that a grain boundary has step-like structure formed by the (111) planes of the adjacent grains in face-centred cubic (FCC) metals. Atoms are emitted from the steps of the shrinking grain into the grain boundary while the steps of the growing grain absorb the same number of atoms out of the grain boundary, leading to GB migration. Ashby^19^ postulated using a bubble model where if sliding is accompanied by migration, the grain boundary structure does not change during the GB motion, whereas Cahn and Taylor^20^ developed a unified approach to study the motion of grain boundaries. It was concluded that the coupled migration and sliding is a general motion phenomenon for most GBs and the coupling factor depends on grain misorientation.

With the advent of more powerful computers, the coupled GB motion has been studied extensively by atomistic simulations, a unique tool with which to understand the dynamic response and evolution of the atomic structure during the coupled GB motion. Bishop Jr *et al*.^21^ studied the dynamics of a high-angle tilt boundary by molecular dynamics and found that atomic motions in a grain boundary were highly cooperative and depended largely on the boundary structure. They explained the coupled GB migration and sliding by a grain boundary dislocation mechanism. Molteni *et al*.^22^ conducted an ab initio simulation of the sliding process at a Σ5(001) twist grain boundary in germanium (Symmetrical coincidence site lattice (CSL) GB are designated as ΣN(h k l), where Σ is the reciprocal density of CSL sites, N is a Σ value and (h k l) is the indices of the GB plane), and found that sliding occurred through a stick-slip mechanism. Chandra and Dang^23^ studied the sliding and migration of [110] symmetric tilt grain boundaries in aluminium under applied displacement and applied force conditions and found that pure grain boundary sliding without migration was caused by applying external displacement, while the coupled migration and sliding was induced by applied force. The former finding was inconsistent with other simulation studies. Ballo and his co-workers^24^,^25^ investigated the effects of temperature and vacancies on the GB sliding and migration and observed that the annealing temperature played an important role in determining the grain boundary energetics and mobility, and the sliding and migration properties partially depended on the position of the vacancy in the GB core. Sansoz and Molinari^26^ studied the mechanical response of symmetric tilt GBs and asymmetric tilt GBs in Cu and Al under simple shear. Here, the deformation of the boundaries was found to operate by three modes, depending on the GB equilibrium configuration: GB sliding by uncorrelated atomic shuffling, nucleation of partial dislocations from the interface to the grains, and GB migration. Zhang and his co-workers conducted molecular dynamics simulations to study elastically driven GB migration^27^, curvature driven GB migration^28^, and shear stress-driven GB migration^29^. Cahn *et al*.^30^ and Suzuki and Mishin^31^ carried out molecular dynamics simulations to investigate the stress-induced migration of [001] symmetrical tilt GBs in copper, and then provided a detailed description of the atomic motions during the coupled GB motion. They found that the GB coupling factor is a geometric constant whose value can be predicted from simple geometric considerations, and they also identified two distinct modes of coupled GB motion. Indeed their predictions on the coupling factors of [001] symmetrical tilt GBs have proven to be in excellent agreement with the subsequent bicrystal experiments^13^. Trautt *et al*.^32^ applied a combination of molecular dynamics and phase field crystal simulations to investigate the stress-driven motion of asymmetrical GBs, and demonstrated that the coupling factor exhibited a non-trivial dependence on the misorientation and inclination angles.

It is widely accepted that the GB coupling factor is a geometric constant that depends only on GB crystallography^33^, but experiments^34^,^35^,^36^,^37^ and atomistic studies^38^,^39^,^40^,^41^,^42^ have demonstrated that for a given set of misorientation and inclination of the boundary plane, the atomic structure of the grain boundary is not necessarily unique. Therefore, there is a strong scientific interest to investigate whether different GB structures with the same GB crystallography could cause different GB behaviours. A multiplicity of GB structures can be induced by segregation and absorption or the emission of point defects^38^. Merkle and Smith^34^ observed the atomic structures of NiO bicrystals near Σ = 5(310) and Σ = 13(510) GBs in a high resolution transmission electron microscopy (HRTEM), and found that: (1) several different grain boundary structures existed for each GB; (2) asymmetric structural units were quite common, even in symmetric GB’s; (3) the Σ13(510) boundary deviated on an atomic scale from a planar configuration in analogy to surface roughening or surface reconstruction. Distinct grain boundary structures (structural multiplicity) were also detected experimentally in Σ9(221) symmetric tilt boundaries in aluminium^35^,^36^ and in Σ17(410) symmetrical tilt boundaries in gold^37^. As an effective alternative, atomistic simulation provides deeper insights into grain boundary structures. Structural multiplicity has been predicted in a number of atomistic simulations^38^,^39^,^40^,^41^,^42^. To the best of the author’s knowledge, the effect of GB structural multiplicity on GB coupled motion has rarely been studied.

In the present study we investigated the structural multiplicity of the symmetric tilt Σ5(310) boundary in aluminium and its influence on GB behaviour by molecular dynamics simulations and found that the GB structural multiplicity did affect the GB behaviour. Three different GB structures were observed in the MD simulations and they were found to be responsible for three types of GB behaviours. A detailed analysis has also been carried out to gain a deeper understanding of the atomistic mechanism of GB coupled motion.

## Results

### Shear coupled GB motion

**Figure 1:**
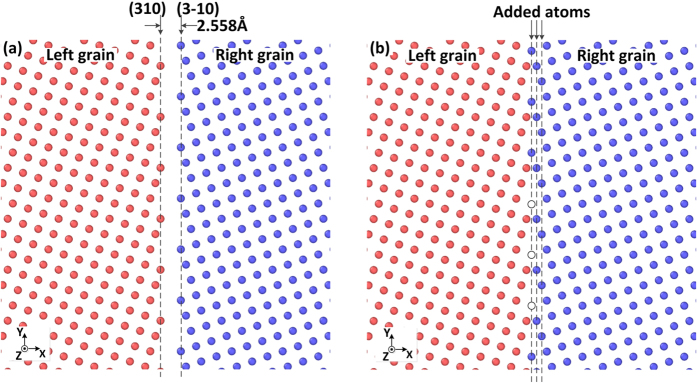
Starting atomic configuration around the GB: (**a**) first starting configuration (SC-1); (**b**) second starting configuration (SC-2).

The grain boundary (GB) studied in this paper was the symmetric tilt Σ5(310) boundary in aluminium, where a bicrystal structure was used in the simulation. The details of model construction was introduced in the *Methods* section. The previous study^39^,^43^,^44^ indicated that distinct GB structures can be achieved by removing or adding different numbers of atomic layers parallel to the boundary in the starting configuration. In the present study two starting atomic configurations were used, as shown in Fig. 1. In the first starting configuration (SC-1), the distance between the furthest right (310) plane of the left grain and the furthest left 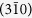 plane of the right grain was 2.558 Å. The second starting configuration (SC-2) was generated by adding three 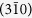 layers of atoms to the right grain of the first starting configuration, as shown in Fig. 1(b). Each layer added consisted of 160 atoms. 100 randomly selected atoms in the left added layer, as marked by the hollow circles in Fig. 1(b), were then deleted.

**Figure 2:**
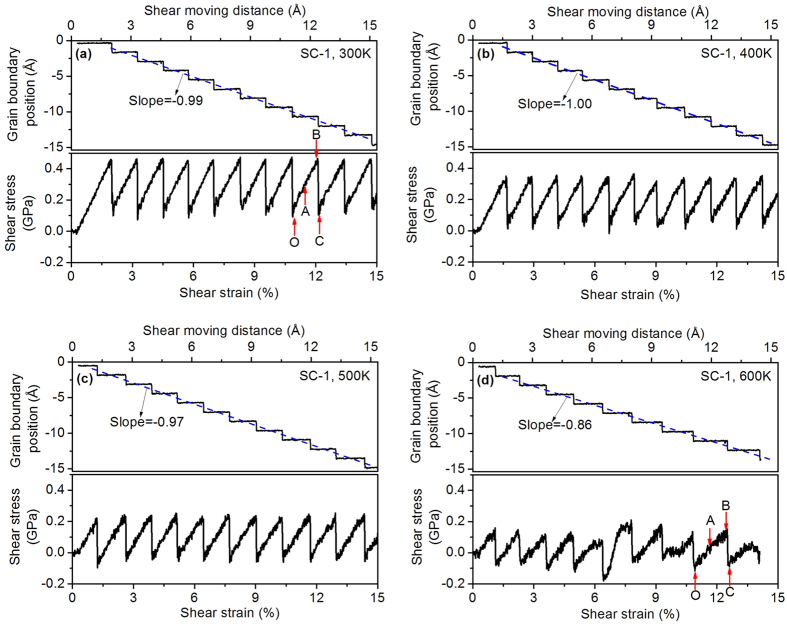
Calculated GB position and shear stress at various temperatures for SC-1: (**a**) 300 K; (**b**) 400 K; (**c**) 500 K; (**d**) 600 K.

Figure 2 shows the grain boundary position in the upper panels and average shear stress in the lower panels as functions of the shear moving distance and the shear strain for the first starting configuration (SC-1). The shear moving distance was calculated by multiplying the applied velocity by the deformation time, while the shear strain was the shear moving distance divided by the width of the simulation cell along the X direction. Figure 2(a–d) correspond to the simulation temperatures of 300 K, 400 K, 500 K, and 600 K, respectively. The shear stress shown in the figures is the average of the shear stresses over all the atoms. A coupled GB motion is clearly observed in the upper panels of the figures. All the GBs move to the negative X direction under the applied shear deformation. At all four temperatures the GBs exhibit in a stop-and-go mechanism. As the shear deformation proceeds the GB position remains unchanged for a period of time (‘stop’ period), but when the increment of shear strain reaches a certain value, the GB suddenly moves (‘go’ period). This GB movement results in a plateau followed by a step in the GB position curve. The blue dashed line in the upper panel of Fig. 2 represents the linear regression line of the GB position curve. The coupling factor (β) of the GB motion can be determined as the slope of the blue dashed line, where β is 0.99, 1.00, 0.97 and 0.86 for T = 300 K, 400 K, 500 K and 600 K, respectively. The calculated coupling factors at 300 K and 400 K are very close to unity, which agrees with the results of experiments conducted for the same misorientation at room temperature^13^, but as the temperature increases to 500 K the coupling factor decreases slightly and then drops suddenly to 0.86 at a temperature of 600 K. Like the stop-and-go mechanism of GB movement, the shear stress is of the stick-slip behaviour, as shown in the lower panels of Fig. 2. During the ‘stop’ period of GB motion, the shear stress increases with the shear strain. An almost linear relationship between the shear stress and shear strain is observed for temperatures from 300 K to 500 K, although a non-linear relationship exists in some stick-slip cycles at 600 K. When the GB moves (‘go’ period), the shear stress suddenly drops from the maximum value to the minimum value. The atomic configurations of the bicrystal simulation model with SC-1 GB at 300 K under shear deformation are shown in Fig. S2(a).

**Figure 3:**
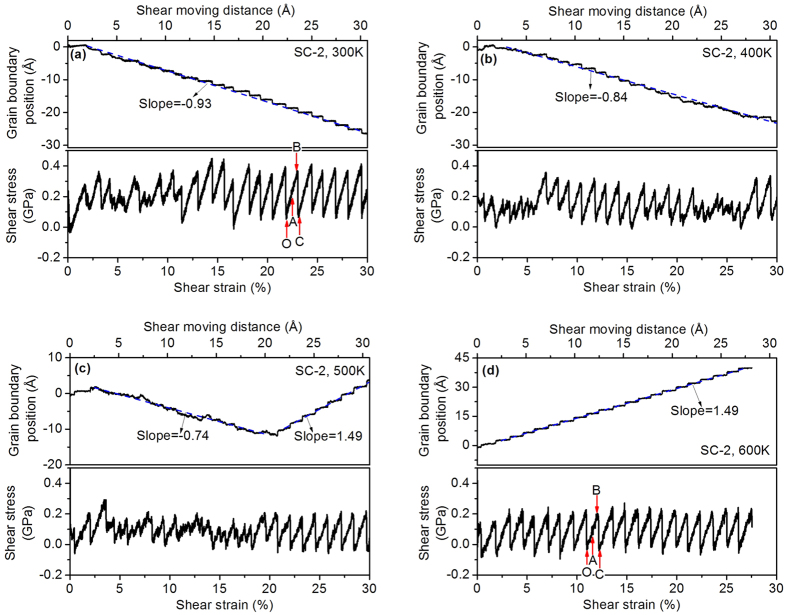
Calculated GB position and shear stress at various temperatures for SC-2 (**a**) 300 K; (**b**) 400 K; (**c**) 500 K; (**d**) 600 K.

Figure 3 shows the calculated GB position and shear stress at four temperatures for the second starting configuration (SC-2). Figure 3(a) shows that the GB moves to the negative X direction, just like the observation in Fig. 2(a). However, the stop-and-go mechanism of the GB movement for SC-2 is not very obvious and the shear stress does not exhibit clear stick-slip behaviour prior to a shear strain of about 11.4%. When the shear strain exceeds 11.4% the stop-and-go GB movement appears in Fig. 3(a) and the shear stress exhibits stick-slip behaviour even though the maximum and minimum shear stresses vary in different stick-slip cycles. The coupling factor is 0.93 at T = 300 K for SC-2, which is lower than SC-1 at the same temperature. The curves of T = 400 K in Fig. 3(b) are generally similar to those in Fig. 3(a). Compared to T = 300 K the coupling factor of T = 400 K decreases to 0.84. When the temperature reaches 500 K a dual GB behaviour can be seen in Fig. 3(c): the GB initially moves to the negative X direction under the imposed shear deformation and then changes to the positive X direction when the shear strain exceeds 20.8%. The first GB behaviour is similar to that observed in Fig. 3(a,b), but in comparison to these figures, the curves of the GB position and shear stress in Fig. 3(c) are more stochastic and the coupling factor has dropped to 0.74. The second GB behaviour observed in Fig. 3(c) exhibits periodicity, which looks similar to that observed in the SC-1 cases (Fig. 2), but a careful inspection indicates that they are not the identical. Unlike the SC-1 cases, the GB after a shear strain of 20.8% in Fig. 3(c) does not maintain a fixed position when the shear stress increases with the shear strain. In another words the GB does not have a fully ‘stop’ period; it continues to move slowly during this period. A GB coupled motion cycle can be divided into a ‘slowly move’ period and a ‘quickly go’ period. The ratio of the GB migration distance during the ‘quickly go’ period to the ‘slowly-move’ period is approximately 2. The dual GB behaviour of SC-2 at 500 K is similar to the experiment observations for [0 0 1] tilt GBs in aluminium^13^, which showed an abrupt transition of the migration mode with the increasing misorientation at room temperature. However, the current study shows that the GB can change its migration mode for a fixed misorientation with the increasing temperature, implying the influence of temperature on the GB structure. The GB behaviour at T = 600 K, as shown in Fig. 3(d), is the same as the second GB behaviour of Fig. 3(c), and they both have the same GB coupling factor (1.49). The atomic configurations of the bicrystal simulation model with SC-2 GB at 500 K and 600 K under shear deformation are shown in Fig. S2(b,c) respectively.

According to the results of Figs 2 and 3, three types of the GB behaviour have been observed. In the first, the GB motion exhibits a periodic stop-and-go mechanism and the shear stress is of regular stick-slip behaviour. The coupling factor is close to unity at lower temperatures and it decreases with the temperature. The SC-1 cases at a temperature range of 300 K–600 K (Fig. 2(a–d)) exhibit this type of GB behaviour. The second type of GB behaviour occurs in the SC-2 cases at T = 300 K and 400 K and the first part of the simulation with SC-2 at T = 500 K. In this type of behaviour the GB motion and shear stress are more stochastic, and its coupling factor is smaller than the first type of GB behaviour at the same temperature. In the first two types of GB behaviour the GB migrates to the negative X direction, but in the third type the GB motion exhibits the slowly-move and quickly-go mechanism and the GB migrates in a direction opposite to the previous two types. The coupling factor of the third type of the GB behaviour is much higher than the other two types. The T = 600 K case with SC-2 and the second part of the SC-2 case at T = 500 K exhibit the third type of GB behaviour.

**Figure 4:**
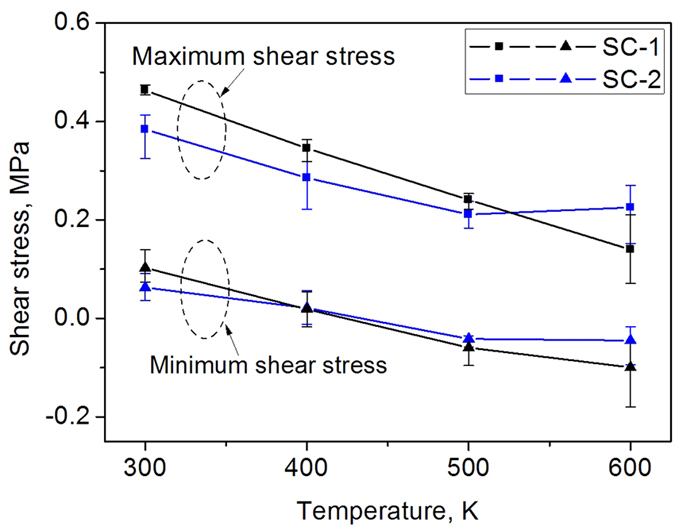
The maximum and minimum shear stress as functions of the simulation temperature.

Figures 2 and 3 show how the shear stress varies between the maximum and minimum values in each GB motion cycle. We calculated the average values and the variations of the maximum and minimum shear stress for all the GB motion cycles and plot them against temperature in Fig. 4. The solid square symbols and solid triangular symbols represent the average values of the maximum and minimum shear stress, respectively. It is clear that the maximum and the minimum shear stress are dependent of the temperature, and in fact decrease with the temperature. All the maximum shear stresses are positive, but the minimum shear stress could be negative at higher temperatures (500 K and 600 K). The SC-1 cases have higher maximum shear stresses than the corresponding SC-2 cases at temperatures up to 500 K, and when T = 600 K, the SC-2 case needs a higher shear stress to migrate the GB. The SC-1 case and the SC-2 case have similar minimum shear stresses at T = 400 K and T = 500 K. The SC-1 case has a higher minimum shear stress than the SC-2 case at T = 300k, they are opposite at T = 600 K. The variations in the maximum and minimum shear stress are represented by the error bars in Fig. 4. Both cases generally have larger variations of shear stresses at T = 600 K, but from T = 300 K to T = 500 K the maximum stress shows larger variation for the SC-2 cases than the SC-1 cases.

### GB structural multiplicity

**Figure 5:**
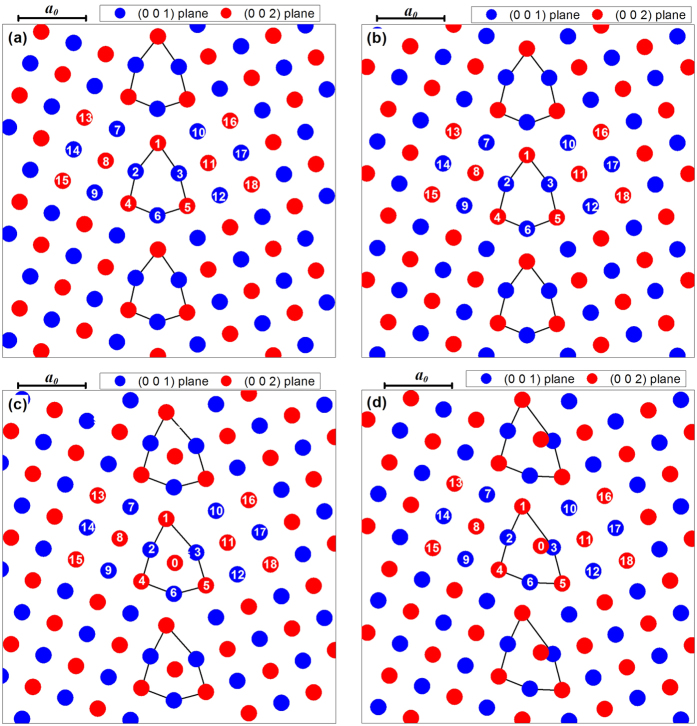
Reconstructed atomic configuration around the GB: (**a**) SC-1 and T = 300 K; (**b**) SC-1 and T = 600 K; (**c**) SC-2 and T = 300 K; (**d**) SC-2 and T = 600 K.

This study aimed to investigate the effect that GB structural multiplicity has on GB behaviour under shear deformation. Two distinct starting GB configurations were intentionally used in the simulations and three types of GB behaviours are shown in Figs 2 and 3. The first question to be answered is: have the two starting GB configurations resulted in different GB structures after thermal relaxation? Figure 5 shows the reconstructed atomic structures around the GBs for four selected simulation cases (SC-1 and SC-2 at T = 300 K and 600 K) after thermal relaxation (at Points O given in Figs 2 and 3). The reconstruction of GB structure is introduced in the *Methods* section. The atoms are observed along the Z direction. Figure 5 only shows the atoms at two successive (001) planes; they are then called the (001) plane and the (002) plane, respectively. The (001) plane and the (002) plane shown in Fig. 5 are blue and red, respectively. Note that all the GBs consist of periodically distributed structural units which are marked as solid lines. With the SC-1 cases (Fig. 5(a,b)), the structural unit has a kite shape and each structural unit is made up by six atoms. Such a structural unit was widely observed in the experiments^45^ and predicted by MD simulations^8^,^38^,^39^, and it has long been recognised as the most stable GB structural unit with the lowest energy for the symmetric tilt Σ5(310) boundary of FCC metals^39^,^45^. This structural unit is called C1 in the following. The constitutive atoms of C1 are labelled 1~6 in Fig. 5(a,b), and the neighbouring atoms of the GB structural unit, namely Atoms 7-18, are also marked in the figures.

Figure 5(c,d) show that the GB structural units of the SC-2 cases consist of seven atoms. Six of them are connected by solid lines, which are analogous to the GB structural units of the SC-1 cases. An extra atom is seen in the midst of these six atoms. The extra atom is located at the core of the structural unit at T = 300 K (Fig. 5(c)), but it is close to the right hand side of the structural unit at T = 600 K (Fig. 5(d)). It should be noted that an extra atom does not exist in all GB structural units, and while there were 160 GB structural units, there were only 60 extra atoms in each simulation cell. The extra atoms were used intentionally to represent the diffused interstitial atoms in the GB. Since their number was less than the number of GB structural units, they can easily be diffused along the Z direction. The GB structural unit in Fig. 5(c) is called C2 in this study. Vitek *et al*.^38^ investigated the multiplicity of GB structures by atomistic simulations and then predicted two GB structures for the tilt Σ5(310) boundary in Cu. They were the same as the C1 and C2 GB structures we obtained.

The shape of the GB structural unit for the SC-2 case at T = 600 K differs from C1 and C2. The GB structural unit in Fig. 5(d) is defined as C3. C3 has a triangular shape, whereas C1 and C2 are kite shaped. In the C3 GB structure, Atom 0 is close to Atom 3, but Atom 0 and Atom 3 are on different (001) planes. Merkle and Smith^34^ observed the bicrystal specimens of NiO with the FCC structure in a high-resolution transmission electron microscopy (HRTEM), and found two different GB core structures (Structure A and Structure B) in the Σ5(310) tilt grain boundary of NiO. The C3 GB structure predicted in the present study was similar to Structure B observed experimentally by Merkle and Smith, but Atom 0 was not shown in the HRTEM image because only fully relaxed structures can be observed in HRTEM. Since Atom 0 has higher energy and is highly unstable, it can travel easily through the core of the GB structure in the Z direction and escape from the GBs near the surface of the HRTEM specimen. Our MD simulations clearly showed that the GB structure is not necessarily unique for a given misorientation. Indeed due to the diffusion of vacancies, interstitials, or segregation GBs with local minimum energy configurations could exist in real materials. The C2 and C3 structural units can be regarded as the C1 structural unit with a diffused interstitial atom (Atom 0) occupying two different locations.

### Effect of shear strain

**Figure 6:**
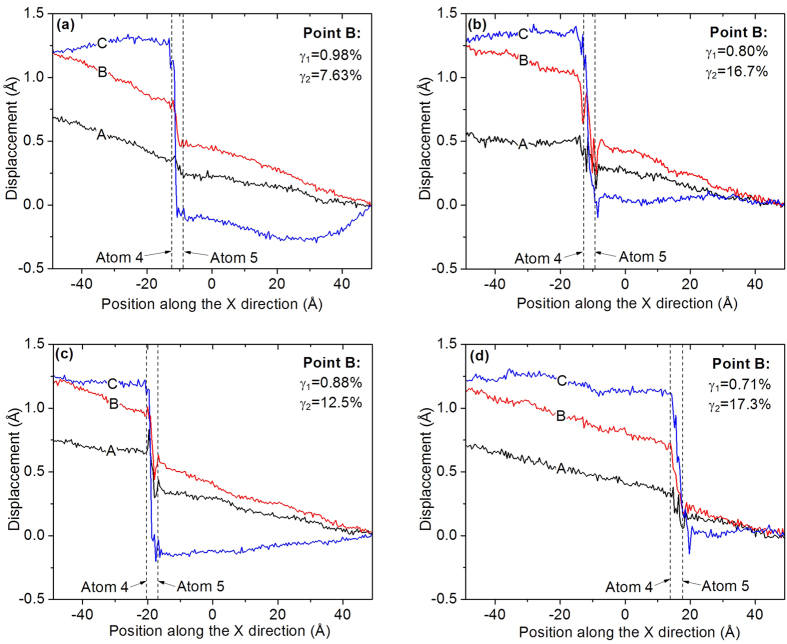
Distribution of the average displacement along the Y direction: (**a**) SC-1 and T = 300 K; (**b**) SC-1 and T = 600 K; (**c**) SC-2 and T = 300 K; (**d**) SC-2 and T = 600 K.

In the MD simulations a shear displacement was applied at the left boundary of the simulation cell. It would be interesting to know how the shear deformation is transferred to the GB and whether the distribution of shear deformation is uniform in the simulation cell. In order to answer the second question, the simulation cell was divided evenly into 250 slabs along the X direction. The average displacement along the Y direction and the average X coordinate were calculated for the dynamic atoms in each slab at Points A, B, and C, which have been marked in Figs 2 and 3. For the displacement calculation, the atomic positions at Point O (marked in Figs 3 and 4) were chosen as references. Figure 6 plots the average Y displacement against the average X coordinate for four cases, and the slope of the displacement curve is the shear strain. The dashed lines give the average X coordinates of Atoms 4 and 5, and also indicate the location of the GB structural unit. Figure 6 shows that the curves of four cases have similar trends. At Point A the displacement curve is almost linear inside two grains and has made an abrupt change around the GB (between two dashed lines). This means that the shear strain in the grains is almost constant but it has a larger value around the GB. At Point B the shear strain inside the grains and around the GB has increased more than at Point A, but the increasing shear strain around the GB is more profound. Once the GB migration is completed, namely at Point C, the displacement curve inside two grains tends to become flat and a very large slope of the displacement curve can be seen around the GB. This occurs because there was a relative slip between two grains inside the GB structural unit and the shear stress in two grains had been released. The average values of the shear strain in grains (γ_1_) and in the GB structural unit (γ_2_) at Point B, corresponding to the maximum shear stress before the GB migration, are given in the figures. They show that γ_2_ is much larger than γ_1_ for all the cases, which indicates that the GB has less resistance to shear deformation than the bulk grains, but as the temperature increases, γ_1_ decreases and γ_2_ increases. A comparison between the SC-1 cases and the SC-2 cases shows that γ_1_ of the SC-1 case is less than the SC-2 case for the same temperature, whereas γ_2_ has an opposite relationship. The detailed process of the shear deformation transferred to GB are presented in the supplementary materials.

## Discussion

**Figure 7:**
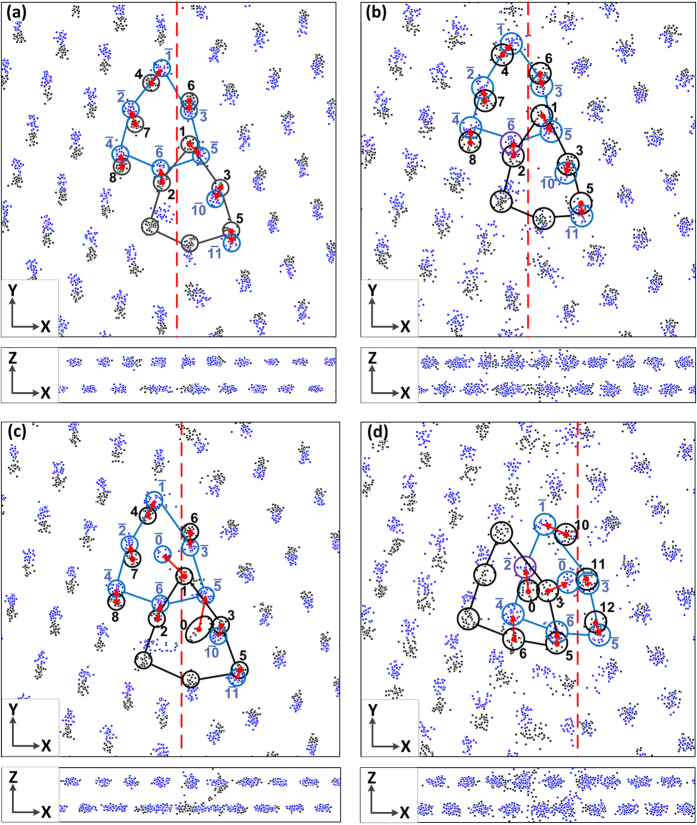
Atomic trajectories during the GB migration: (**a**) SC-1 and T = 300 K; (**b**) SC-1 and T = 600 K; (**c**) SC-2 and T = 300 K; (**d**) SC-2 and T = 600 K.

In this study, we observed three GB structures (C1, C2 and C3) in Fig. 5 and three types of GB behaviours in Figs 2 and 3. It is clear that different GB structures are responsible for different types of GB behaviours, so in this section we will discuss the atomistic mechanism of GB migration. Figure 7 shows the atomic positions at the multiple simulation times with an interval of 3 ps for a period of 180 ps. The X-Y plane view is shown in the upper panel and the X-Z plane view in the lower panel. The black and blue dots represent the atomic positions before and after the GB migration, respectively. The black circles and blue circles cover most of the positions of the atoms (concentrations of the atomic positions) before and after the GB migration, respectively. The red arrows link the centres of the black and blue circles, and also indicate the atomic displacement vector. Figure 7 shows the movements of the atoms from their positions in one grain, before GB migration, to their new positions in another grain after GB migration. For the sake of brevity, the GB structures before and after their migration are called the old GB structure and the new GB structure, respectively.

Figure 7(a,b) show the results of the SC-1 cases where T = 300 K and T = 600 K, respectively, and indicate how the atomic trajectories are similar to each another. The only difference between two simulations is that the area where the atoms concentrate at T = 600 K is larger due to the stronger thermal fluctuation at higher temperature. In these two cases, when the GB migrates the atoms in the left grain jump up and the atoms in the right grain move down. The atoms in and near the old GB structure are labelled by black numbers and those in and near the new GB structure by blue numbers with a bar on the top of each number. The old GB and new GB both consist of C1 structural units. Like the internal atoms of the left grain, Atoms 2 and 8 move straight up by an average distance of about 1.36 Å during the GB migration period to become Atoms 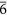 and 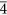, respectively, in the new GB structure. Atom 7 has a similar upward movement with a slight bias to the left, where it forms Atom 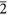. Atoms 3, 5, and 6 tend to coincide with the internal atoms of the right grain and they still evolve to Atoms 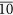, 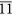 and 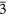 in the new GB structure, respectively. Atoms 1 and 4 have special movements; Atom 1 moves to the lower right to form Atom 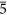 while Atom 4 proceeds to the upper right to become Atom 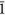. A dashed red line is drawn to separate the atoms moving up from the atoms moving down during the abrupt GB migration, and to also indicate an interface where two grains have a relative sliding. The lower panels of Fig. 7(a,b) show that all the atoms vibrate at their own (001) planes, but at T = 600 K (Fig. 7(b)) the atoms have larger amplitude of vibration. Indeed, some atoms could jump to their neighbouring (001) planes due to thermal activation.

For the SC-2 case at T = 300 K (Fig. 7(c)), most atoms move like their counterparts in the SC-1 cases, apart from Atom 0 and Atom 1. In the SC-1 cases Atom 1 moves to the lower left to form Atom 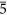 but in the SC-2 case at T = 300 K, Atom 1 jumps into the core of the new GB structural unit and becomes Atom 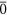, while Atom 0 evolves into Atom 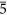 subjected to a upward movement. The dashed red line indicates the sliding interface between two grains. A comparison between the old GB structural unit and the new GB structural unit indicates they have different local structures; the new GB structural unit in Fig. 7(c) is the same as the C2 structural unit identified in Fig. 5, while the old GB structural unit was similar to the C3 structural unit. This means that after GB migration the extra atom (Atom 0) was located in the core of the GB structure, but as shear deformation proceeds Atom 0 tends to occupy the site near Atom 3 (Atom 0 site in C3). Both positions are metastable and have higher potential energies. During the shear deformation, Atom 0 could frequently move between two positions, but this switch of position also affects the positions of the other surrounding atoms. It should be noted that the GB position was calculated as the average X coordinates of all the disordered atoms around the GB, and therefore this frequent switch of position by Atom 0 induces stochastic GB positions, which is why we did not see an obvious stop-and-go behaviour for the SC-2 case at T = 300 K in Fig. 3(a). When Atom 0 is located close to Atom 3 (in the old GB structure), it can jump to another (001) plane and swap positions with Atoms 3. While Atom 0 can push one of two neighbouring Atoms 3 away and occupy this position, another Atom 3 may fill up the gap left by Atom 0. This behaviour is indicated by the black dots between two (001) planes in the lower panel of Fig. 7(c).

Figure 7(d) is indicative of the SC-2 case at T = 600 K where the internal atoms of the left grain move up and those of the right grain move down slightly. The magnitudes at which these atoms move in the two grains differ in that the former is larger than the latter. Atoms 1, 2, and 4 in the old GB structure have similar movement to the internal atoms of the left grain, where Atoms 0, 5, and 6 jump upwards over different distance. Atoms 11 and 12 have a similar movement to the atoms inside the right grain but Atoms 10 and 3 had special movements, such that Atom 10 moves to the upper left and Atom 3 moves to the upper right. Atoms 3, 10, 0, 11, 6, 12 and 5 in the old GB structure evolve into Atoms 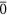
–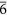 in the new GB structure via a GB migration. The dashed red line in Fig. 7(d) indicates the sliding interface between two grains and also shows that the old GB and the new GB, both of which consist of C3 structural units. The lower panel of Fig. 7(d) shows that Atoms 0 and Atoms 3 switch positions frequently in the old GB structure, whereas Atoms 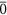 and Atoms 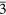 had similar behaviour in the new GB structure. This is shown by the black and blue dots between the two (001) planes in Fig. 7(d).

Table S1 lists the potential energies of key atoms in the old GB structure and the new GB structure for the four simulation cases. The potential energies given in the table were calculated at Point B for the old GB structure and at Point C for the new GB structure, respectively. Here the potential energies of some atoms decrease after GB migration, while those of other atoms increase. The atoms with the highest potential energy are Atom 2 for SC-1 at both T = 300 K and T = 600 K, Atom 0 for SC-2 at T = 300 K, and Atom 3 for SC-2 at T = 600 K, respectively. These atoms play a key role in the coupled GB motion.

In conclusion, we investigated the structural multiplicity of the symmetric tilt Σ5(310) boundary in aluminium and its influence on the behaviour of the GB at a temperature range of 300 K–600 K by molecular dynamics simulations. Two starting atomic configurations (SC-1 and SC-2) were adopted in the simulations which led to three different GB structures (C1, C2 and C3) after thermal relaxation. The three GB structures induced three types of GB behaviours, all of which clearly demonstrated that the structural multiplicity of GB did affect its behaviour. The atomic mechanism responsible for the behaviour of each GB has been analysed in detail. The result of this study can provide further information to understand the mechanical behaviour and the grain boundary mediated plasticity in nanocrystalline materials.

## Methods

### Model construction

For constructing the bicrystal model with Σ5(310) grain boundary, two identical aluminium crystals were initially aligned with the coordinate system with [100], [010] and [001] parallel to the X, Y, and Z axes respectively^46^. The lattice constant was *a*_*0*_ = 4.05 Å. One crystal was then rotated around the Z axis (tilt axis) by θ/2 (θ = 36.87°) in a clockwise direction, while the other crystal was rotated around the same axis by θ/2 in an anticlockwise direction. The GB plane was set parallel to the Y-Z plane such that the first crystal formed the left grain and the second crystal formed the right grain. The GB plane was the (310) crystallographic plane in the left grain and the 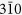 plane in the right grain, as shown in Fig. S1. The dimensions of the simulation cell along the X, Y, and Z axes were 102.3 Å, 102.3 Å and 40.5 Å, respectively.

### Molecular dynamics simulation

There was a slab of fixed atoms on the left hand side and the right hand side of the simulation cell, and the slab was 1.279 Å thick along the X direction. During the simulation, the fixed atoms were frozen in their perfect lattice positions relative to one another, but they still imposed inter-atomic forces on their neighbouring dynamic atoms. During the MD simulations, the bicrystal system was thermally relaxed at the corresponding simulation temperature for 50 ps and then a shear parallel to the GB was applied by moving all the fixed atoms from the left grain at a constant velocity of V = 0.5 m/s, while the fixed atoms from the right grain remained motionless. Simulation temperatures of 300 K, 400 K, 500 K, and 600 K were selected in this study to cover the range of interest. A molecular dynamics code LAMMPS was used in all the simulations. The interatomic potential adopted for Al was the embedded-atom method (EAM) potential of Mishin *et al*.^47^ that was developed by fitting a large set of experimental and *ab initio* databases. This EAM potential could accurately reproduce the basic equilibrium properties of Al, the elastic constants, the phonon-dispersion curves, the vacancy formation and migration energies, the stacking fault energies, and the surface energies^47^. The MD simulations were performed in the isothermal-isobaric (NPT) ensemble (Nose–Hoover thermostat) with zero pressure in Y and Z directions. The equations of atomic motion were integrated using the velocity Verlet algorithm with an integration time step of 1 fs. The stress components were calculated using the expression taken from the Virial theorem, and the average atom volume was used in the stress calculations^48^.

### GB reconstruction and tracing

The common neighbour analysis (CNA) pattern was calculated for all atoms at each storing interval. The CNA pattern is a useful measure to characterise the type of local lattice structure. There are five CNA patterns, numbered from 1 to 5, that correspond to FCC lattice, hexagonal close packed (HCP) lattice, body-centred cubic (BCC) lattice, icosahedral lattice, and unknown lattice. Most atoms around the GB and some atoms near the thermally activated vacancy at high temperature were categorised into unknown lattices, namely CNA = 5. A histogram for the X coordinates of all CNA = 5 atoms was plotted and then fitted by a single-peak Gaussian distribution function for each stored simulation time step. The peak of the fitted distribution function gave the average position of the GB. Particularly, it is always difficult to display the motion pattern of an atom in the MD simulations of higher temperatures because the higher frequency motion of the atom has been disturbed. The common approach used to solve this problem is to calculate the time averages of each atomic coordinate over a number of simulation time steps^2^,^21^, but it may smooth out any sudden atomic movement. In this study we traced the atomic movements in a different way. Here the GB used in our MD simulations consisted of about 160 GB structural units, and since all the structural units were distributed periodically, we first moved all the structural units and their neighbouring atoms to a pre-defined position according to the known period relationship, and then calculated the average of the coordinates of the atoms located at the same local position to obtain the equivalent local coordinates, and then formed an equivalent structural unit. To help facilitate the analysis, the simulation cell was reconstructed by the equivalent structural units through the known periodic relationship.

## Additional Information

**How to cite this article**: Cheng, K. *et al*. Coupled grain boundary motion in aluminium: the effect of structural multiplicity. *Sci. Rep*. **6**, 25427; doi: 10.1038/srep25427 (2016).

## Supplementary Material

Supplementary Information
