# Supplementary material for: Coupled grain boundary motion in aluminium: the effect of structural multiplicity
Source: Sci Rep. 2016 May 3;6:25427. doi: 10.1038/srep25427 (PMC4853718; doi:10.1038/srep25427)
Supplement: Supplementary Information [file srep25427-s1.pdf]

Supplementary Materials

Coupled grain boundary motion in aluminium: the effect of structural multiplicity

Kuiyu Cheng[†], Liang Zhang[†], Cheng Lu[*], Kiet Tieu

School of Mechanical, Materials and Mechatronic Engineering, University of Wollongong, Wollongong, NSW 2522, Australia

(1) Fig.S1 - Schematic of the bicrystal simulation model.

(2) Fig.S2 - Snapshots of the bicrystal simulation model at different shear stages

(3) Table.S1 - Potential energies of the key atoms before and after GB migration.

(4) Fig.S3 - Atomic positions with marked shear units around the GB

(5) Mechanism of shear deformation transferred to GB.

[†]The two authors contributed equally to this work.

*Corresponding author. Tel.:+6142214639; fax:+61242213101;

E-mail address: chenglu@uow.edu.au (C. Lu).

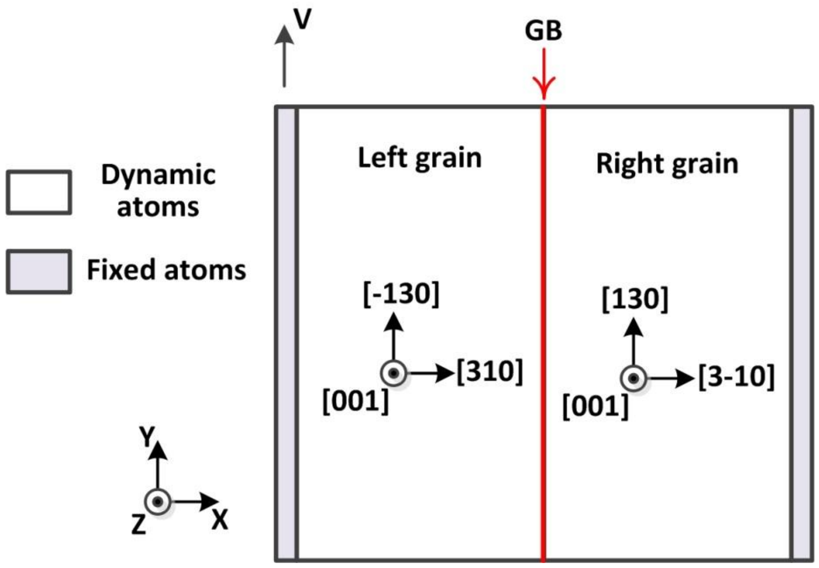

Fig.S1 Schematic of the bicrystal simulation model with a symmetric tilt Σ5(310) grain boundary.

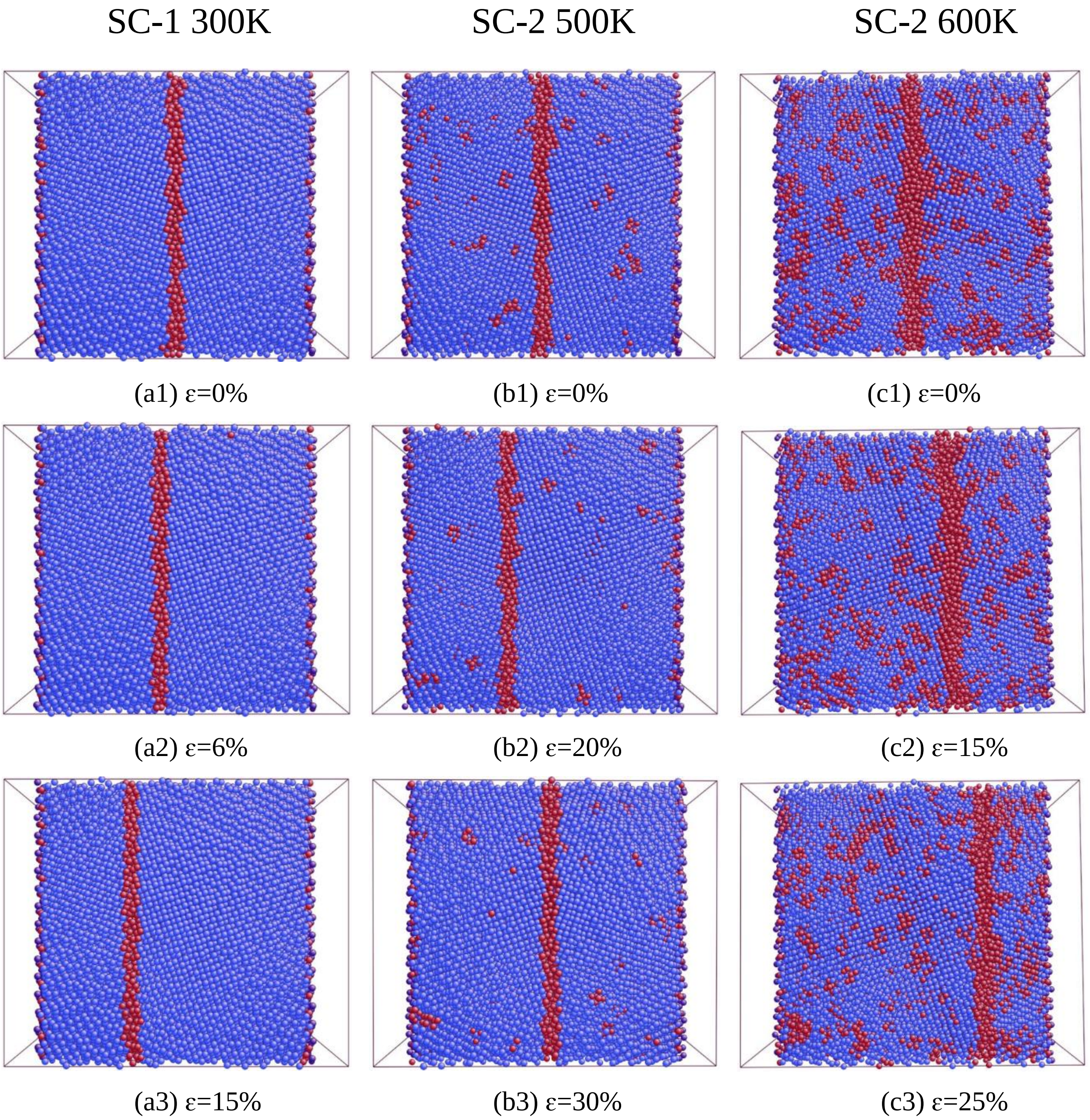

Fig.S2 Snapshots of the bicrystal simulation model at different stages under shear deformation. (a1-a3) SC-1 and T=300K; (b1-b3) SC-2 and T=500K; (c1-c3) SC-2 and T=600K. Atoms with perfect fcc structures are colored blue, while atoms at GB area and the disordered atoms caused by higher temperature are colored red.

Table.S1 Potential energies of the key atoms before and after GB migration.

Simulation cases	Before GB migration (Point B)		After GB migration (Point C)		Change in potential energy (eV)
	Atom id	Potential energy (eV)	Atom id	Potential energy (eV)	
SC-1 T=300K	1	-3.298	$\bar{5}$	-3.313	0.015
	2	-3.184	$\bar{6}$	-3.211	0.027
	3	-3.206	$\overline{10}$	-3.336	0.130
	4	-3.308	$\bar{1}$	-3.298	-0.010
	5	-3.316	$\overline{11}$	-3.344	0.028
	6	-3.204	$\bar{3}$	-3.195	-0.009
	7	-3.306	$\bar{2}$	-3.189	-0.117
	8	-3.338	$\bar{4}$	-3.312	-0.026
SC-1 T=600K	1	-3.245	$\bar{5}$	-3.260	0.015
	2	-3.151	$\bar{6}$	-3.166	0.015
	3	-3.172	$\overline{10}$	-3.263	0.091
	4	-3.254	$\bar{1}$	-3.241	-0.013
	5	-3.260	$\overline{11}$	-3.283	0.022
	6	-3.155	$\bar{3}$	-3.152	-0.003
	7	-3.247	$\bar{2}$	-3.163	-0.083
	8	-3.279	$\bar{4}$	-3.261	-0.019
CS-2 T=300K	0	-3.160	$\bar{5}$	-3.180	0.020
	1	-3.232	$\bar{0}$	-3.228	-0.004
	2	-3.242	$\bar{6}$	-3.261	0.019
	3	-3.236	$\overline{10}$	-3.312	0.076
	4	-3.290	$\bar{1}$	-3.231	-0.059
	5	-3.294	$\overline{11}$	-3.329	0.036
	6	-3.252	$\bar{3}$	-3.228	-0.024
	7	-3.313	$\bar{2}$	-3.237	-0.076
	8	-3.337	$\bar{4}$	-3.299	-0.038
CS-2 T=600K	0	-3.167	$\bar{2}$	-3.222	0.055
	1	-3.240	$\bar{7}$	-3.285	0.044
	2	-3.250	$\bar{8}$	-3.305	0.055
	3	-3.147	$\bar{0}$	-3.158	0.011
	4	-3.309	$\bar{9}$	-3.314	0.005
	5	-3.218	$\bar{6}$	-3.211	-0.007
	6	-3.223	$\bar{4}$	-3.297	0.074
	10	-3.235	$\bar{1}$	-3.214	-0.020
	11	-3.212	$\bar{3}$	-3.168	-0.044
	12	-3.288	$\bar{5}$	-3.223	-0.065

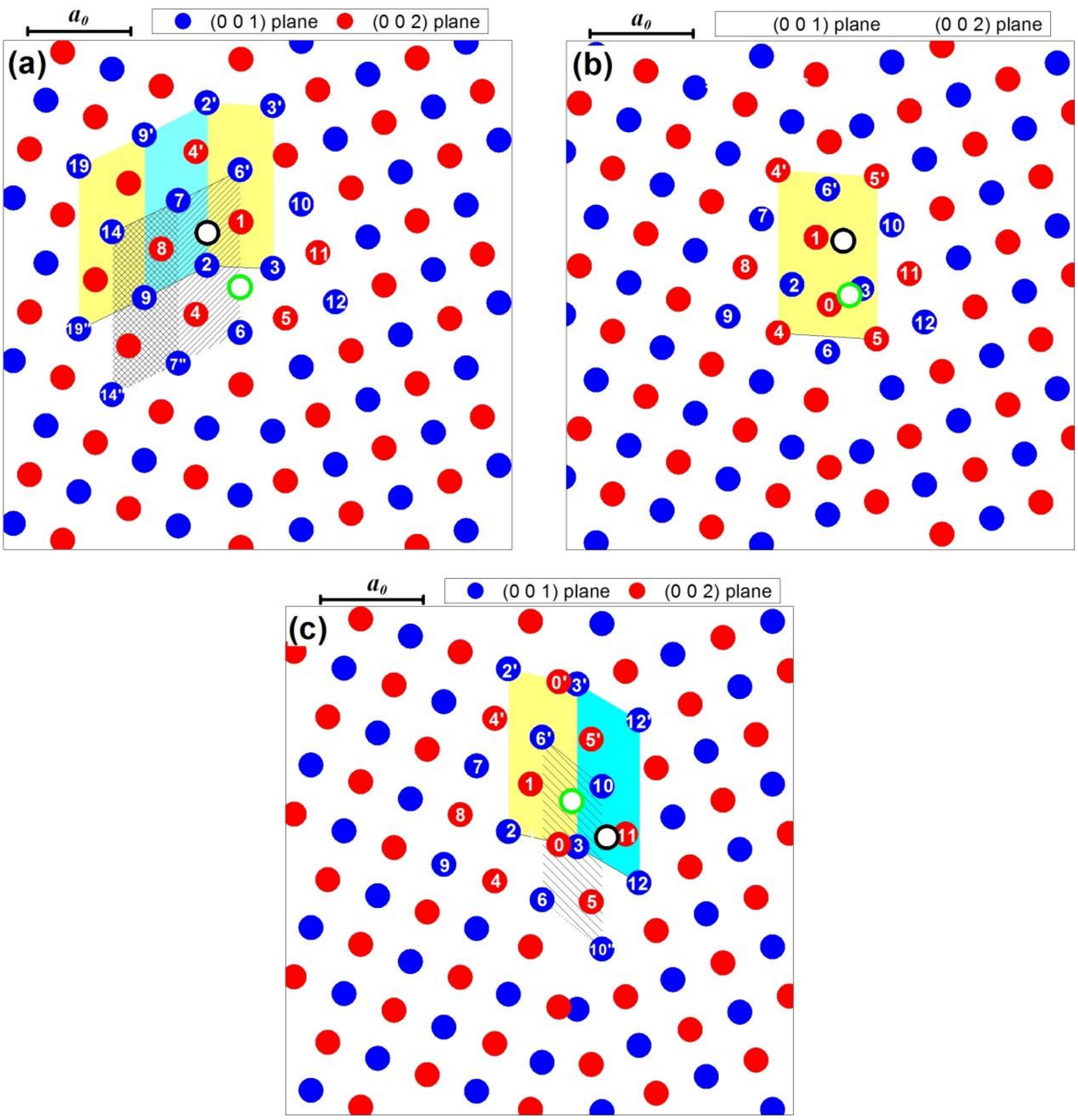

Fig.S3 Atomic positions with marked shear units around the GB: (a) C1 GB structure; (b) C2 GB structure; (c) C3 GB structure.

Mechanism of shear deformation transferred to GB

An analysis of all the simulation cases showed the atoms around the GB tend to occupy three metastable sites: 1) the parent lattice site of the left grain; 2) the parent lattice site of the right grain, and 3) the symmetric position of two grains. In the C1 structural unit, as seen in the SC-1 cases at T=300K and 600K, Atoms 2 and 4 are close to the parent lattice sites of the left grain, Atoms 3 and 5 are near the parent lattice sites of the right grains, and Atoms 1 and 6 are at the symmetric positions of two grains. In the C2 structural unit Atoms 1-6 have the same local positions as those in the C1 structural unit, but the extra atom, Atom 0, is mostly at the symmetric positions of two grains. In the C3 structural unit, Atom 0 and Atoms 1 are at sites close to the parent lattice sites of the left grain.

In order to analyse the transmission of the shear deformation in the simulation cell, we consider several structural units on the (001) plane in the left grain and around the C1 GB; they are shown as shaded in Fig.S3(a) and will be called the shear units. The first shear unit consists of Atoms 9, 9′, 19 and 19″ and the second unit consists of Atoms 7″, 7, 14 and 14″. Both of them are shaped like a parallelogram. Atom 14 is at the centre of the first shear unit and at the corner of the second shear unit. If we ignore the thermal activity, Atom 14 will be always located at the symmetric centre of parallelogram 9-9′-19-19″ during deformation, which will give Atom 14 a stable condition with the lowest energy. When the shear deformation is transferred to the shear unit 9-9′-19-19″, Atoms 19 and 19″ will move up relative to Atoms 9′ and 9 and induce a shear strain in this unit. Since Atom 14 is firmly constrained by Atoms 9, 9′, 19 and 19″, the movement of Atoms 19 and 19″ will drag Atom 14 up. This will indicate that the shear deformation has been transferred from the first shear unit to the second shear unit. In the same manner the shear deformation in the second shear unit will move its central atom (Atom 9) upwards, and in turn transfer the shear deformation to the next shear unit (2-2′-9′-9). Since the shear units inside the grains possess a perfect lattice structure without any defect, they are tightly interlocked with their neighbouring units. The atom at the centre of each shear unit acts like an interlock pin. Interlocking the shear units means that the shear strain can be distributed uniformly inside the grains under the applied shear deformation, as observed in Fig.6.

The shear unit 3-3′-2′-2 is the unit on the C1 GB and the shear unit 6-6′-7-7″ is the unit linking the interior shear unit 2-2′-9′-9 and the GB shear unit 3-3′-2′-2. Unlike the shear units in the grains, there

is no stable central position for these two units because the atoms around the GB are disturbed. This means the interlock pin atoms of these two shear units (Atom 2 and Atom 6′) can only occupy metastable positions and in fact may move from one metastable position to another under the influence of shear deformation. The interlocking of shear units 3-3′-2′-2 and 6-6′-7-7″ is not as strong as the shear units inside the grains, so the GB exhibits less resistance to shear deformation and has larger shear strain than the bulk grains, as observed in Fig.6.

Atom 2 inside shear unit 6-6′-7-7″ tends to occupy a site close to the parent lattice site of the left grain. Table.S1 shows that Atom 2 has the highest potential energy in the entire system, and when the shear deformation is transmitted to the shear unit 6-6′-7-7″, Atoms 7 and 7’ move up and Atom 2 could not fully keep step with Atoms 7 and 7′. As the deformation proceeds the potential energy of Atom 2 increase but when it exceeds the energy barrier Atom 2 will move to other metastable positions. Atom 2 has already occupied a site close to the parent lattice site of the left grain. Under shear deformation, it will move to other types of metastable positions, that is, either a parent lattice site of the right grain or a symmetric position between grains. The core of the GB structure unit, schematically marked by a green circle in Fig.S3(a), is a symmetric position of two grains, but this position is situated at the lower right side of Atom 2. Since Atom 2 is dragged upwards by Atoms 7 and 7′, it is impossible for Atom 2 to occupy this core position. A parent lattice site of the right grain, marked by a black circle in Fig.S3(a), is the only possible metastable position that can be occupied by Atom 2. When it overcomes the energy barrier, Atom 2 can jump from the old metastable position to the new metastable position to become Atom $\bar{6}$ in the new GB structure. During this period it will release its potential energy which will help other atoms overcome their energy barriers. Atom 1 is initially located at the symmetric position, but when Atom 2 moves it pushes Atom 1 to occupy a parent lattice site of the right grain such that Atom 1 becomes Atom $\bar{5}$ in the new GB structure. As Atom 2 moves from its old metastable position to its new metastable position, the connection between Atom 2 and the atoms to its left are weakened considerably, which results in an instantaneous disconnection between two grains. Therefore, we observed a relative slip between two grains at the interface, which are marked in Fig.6. This instantaneous disconnection also releases the elastic energy stored in both grains, which leads to a sudden drop in the shear stress and a release of the shear stress at Point C, as shown in Fig.2(a) and Fig.6(a), respectively. As the atoms around the

GB proceed to their new positions, they will build new connections with surrounding atoms and form a new GB structure.

Fig.S3(b) shows the atomic positions with a marked shear unit on the (002) plane around the C2 GB for the SC-2 case at T=300K. The shear unit 5-5′-4′-4 coloured yellow in Fig.S3(b) is a unit around the GB in which Atoms 0 and 1 are located. Table.S1 shows that Atom 0 has the highest potential energy, and as the shear deformation proceeds it tends to move to two other possible metastable positions; one is schematically marked by a green circle, which is the parent lattice site of the left grain, and the other position, marked by a black circle, is the parent site of the right grain. If Atom 0 occupies the first position (green circle), it will easily swap its position with Atom 3, as can be seen in Fig.6(c). This does not result in any GB migration. When the potential energy of Atom 0 exceeds its energy barrier, it could move to the second metastable position (black circle) and become Atom $\bar{5}$ in the new GB structure, while Atom 0 also pushes Atom 1 to occupy the core of the new GB structure and Atom 1 then becomes Atom $\bar{0}$. As the consequence of above collective atomic movements, the GB migrates to a negative X position, as shown in Fig.3(a). When Atom 0 occupies the green circled position in Fig.S3(b), the GB structural unit looks similar to the C3 GB structural unit, but they have different shapes. The former is shaped like a kite while the latter looks like a triangle. When the simulation temperature of the SC-2 case increases to 500K the initial GB structure unit is the same as that of 300K. However, when the shear deformation reaches a certain level, the thermal energy may trigger a transformation from the C2 structure to the C3 structure which is responsible for the dual behaviour observed at T=500K.

Fig.S3(c) shows the atomic positions with marked shear units on the (001) plane around the C3 GB for the SC-2 case at T=600K. In this case Atom 3 has the highest potential energy and it is located inside the shear unit 5-5′-6′-6 and occupies the site close to the parent site of the right grain. Under shear deformation Atom 3 tends to move to two possible metastable positions; the first position is schematically marked by the green circle and the second one is marked by the black circle respectively. Both of them are the parent sites of the left grain. If Atom 3 moves to the first position, it will be very close to Atom 10, which is not energy preferred, so Atom 3 tends to occupy the second position and push Atom 10 to occupy the first position. This results in a GB migration to the positive direction, as can be seen in Fig.3(d).
